# Structural and Functional Interactions between Transient Receptor Potential Vanilloid Subfamily 1 and Botulinum Neurotoxin Serotype A

**DOI:** 10.1371/journal.pone.0143024

**Published:** 2016-01-08

**Authors:** Xiaqing Li, Julie A. Coffield

**Affiliations:** 1 Department of Pathophysiology, Shanxi Medical University, Taiyuan, Shanxi, China; 2 Department of Physiology and Pharmacology, University of Georgia, Athens, Georgia, United States of America; Southern Illinois University School of Medicine, UNITED STATES

## Abstract

**Background:**

Botulinum neurotoxins are produced by *Clostridium botulinum* bacteria. There are eight serologically distinct botulinum neurotoxin isoforms (serotypes A–H). Currently, botulinum neurotoxin serotype A (BoNT⁄A) is commonly used for the treatment of many disorders, such as hyperactive musculoskeletal disorders, dystonia, and pain. However, the effectiveness of BoNT⁄A for pain alleviation and the mechanisms that mediate the analgesic effects of BoNT⁄A remain unclear. To define the antinociceptive mechanisms by which BoNT/A functions, the interactions between BoNT⁄A and the transient receptor potential vanilloid subfamily 1 (TRPV1) were investigated using immunofluorescence, co-immunoprecipitation, and western blot analysis in primary mouse embryonic dorsal root ganglion neuronal cultures.

**Results:**

1) Three-week-old cultured dorsal root ganglion neurons highly expressed transient TRPV1, synaptic vesicle 2A (SV2A) and synaptosomal-associated protein 25 (SNAP-25). SV2A and SNAP-25 are the binding receptor and target protein, respectively, of BoNT⁄A. 2) TRPV1 colocalized with both BoNT⁄A and cleaved SNAP-25 when BoNT⁄A was added to dorsal root ganglia neuronal cultures. 3) After 24 hours of BoNT⁄A treatment (1 nmol⁄l), both TRPV1 and BoNT⁄A positive bands were detected in western blots of immunoprecipitated pellets. 4) Blocking TRPV1 with a specific antibody decreased the cleavage of SNAP-25 by BoNT⁄A.

**Conclusion:**

BoNT/A interacts with TRPV1 both structurally and functionally in cultured mouse embryonic dorsal root ganglion neurons. These results suggest that an alternative mechanism is used by BoNT⁄A to mediate pain relief.

## Introduction

Botulinum neurotoxins (BoNTs), which are known to be some of the most poisonous substances in existence, are the agents responsible for the fatal disease botulism. Eight serotypes of BoNT (A, B, C, D, E, F, G and H) have thus far been identified [1]. In addition to its extreme toxicity and high potential for inducing morbidity and mortality via its flaccid paralyzing effects on respiratory muscles, BoNT⁄A is also effective as a treatment in a variety of clinical and cosmetic conditions ranging from painful dystonias to facial wrinkles, all based on its ability to block the release of neurotransmitters [2,3]. Among BoNTs, serotype A (BoNT⁄A) is the most common serotype used in a clinical setting. The analgesic effects of BoNT⁄A have generally been attributed to its ability to block the release of pain-related neuropeptides. It has been reported that BoNT⁄A functions by inhibiting the release of calcitonin gene-related peptide(CGRP) from afferent terminals of sensory neurons located in the dorsal root ganglia (DRGs) and trigeminal ganglia [4, 5]. Recently, it was also found that BoNT⁄A decreases the sensitivity of nociceptors in muscles to mechanical stimuli [6]. The sensation of pain is believed to be mediated by various complicated pathways, from the onset of nociceptor activation to the transduction of nociception and, finally, to the sensation of pain in the brain. Therefore, the analgesic properties of BoNT⁄A are complex, and the exact anti-nociceptive mechanism by which BoNT⁄A acts has not yet been completely defined. Thus, a fuller understanding of this mechanism requires further study.

Generally, pain is divided into nociceptive and pathological pain. Nociceptive pain is caused by the sustained activation of peripheral nociceptors in response to peripheral tissue injury. Transient receptor potential vanilloid subtype 1 (TRPV1), also known as the capsaicin receptor, is a typical representative of nociceptors that are expressed predominantly by peripheral sensory neurons and also in some areas of the central nervous system [7]. TRPV1 belongs to the non-selective excitatory cation channels and is a member of the TRP superfamily V, which is mainly involved in the initiation of nociceptive signal transduction upon its activation [8,9]. In addition to capsaicin, TRPV1 is activated by a variety of noxious signals, including high temperature (>43°C), acidic pH (< 5.5), and inflammatory second messengers, such as bradykinin, ATP, and prostaglandins[10]. However, overexpression or hyperactivation of TRPV1 can induce local de–innervation and analgesia [11]. Therefore it is widely thought that TRPV1 acts as a key nociceptor and integrator. Moreover, it has also been reported that TRPV1 is involved in synaptic transmission, in which it modulates neurotransmitter release, plasticity and vesicle recycling [9,12]. More interestingly, BoNT⁄A, when locally injected into the urinary bladder subepithelium, modulates the expression of TRPV1 and relieves detrusor muscle hyperactivity [13,14]. The analgesic and nocifensive effects of BoNT⁄A are evidenced by its ability to directly antagonize the TRPV1 agonist (capsaicin) as well as decrease the expression of TRPV1 [15,16]. However, whether there is some direct relationship structurally or functionally between BoNT⁄A and TRPV1 is currently unclear.

In the present study, we investigated interactions between TRPV1 and BoNT⁄A using co-immunoprecipitation and immunofluorescent colocalization assays. The ability of TRPV1 to block the action of BoNT⁄A was also examined.

## Materials and Methods

This study was conducted using dorsal root ganglion sensory neurons isolated from 30 mouse embryos at embryonic day 15 (E15). All animals were euthanized via inspiration of CO_2_. All experiments were approved by the animal research committees of both the University of Georgia and Shanxi Medical University.

### Isolation and culture of primary embryonic mouse DRG neurons

The procedures for isolating and culturing DRG neurons have been described previously [17,18], and these procedures were followed with some modifications. Briefly, timed–pregnant NIH Swiss mice (G15) were euthanized with CO_2_. The DRGs were dissected from the embryos and placed in a tube containing Hibernate–E solution (BrainBits LLC, USA) at 4°C. The DRGs were then incubated in Krebs buffer containing 0.05% trypsin for 30 min at 37°C. The digestion was terminated by the addition of 0.2% trypsin inhibitor (Sigma, USA) and 0.02% DNase.The solution was then triturated gently until a cloudy suspension appeared. The cells were washed once with Krebs buffer and then resuspended in growth medium that consisted of Dulbecco’s Modified Eagle’s Medium (DMEM, Sigma, USA) supplemented with 2 mM glutamine, 10% fetal bovine serum (FBS), 1% penicillin and streptomycin, 100 ng⁄ml 7S nerve growth factor (Sigma, USA), 80 μM 5-fluoro-2′–deoxyuridine, and 100 μM uridine. Approximately 0.2–0.3×10^5^ cells⁄ml were placed on polylysine–coated coverslips. The cells were grown at 37°C in an atmospheric environment containing 95% O_2_ and 5% CO_2_, and the media was refreshed every 3 days.

### Immunofluorescent (IF) staining and colocalization

DRG cells grown on coverslips were cultured for 1–3 weeks before harvesting. At different time points, the cells were fixed with 4% paraformaldehyde for 1 hour. A solution of 0.1% Triton X–100 was added to the cells after fixation to increase membrane permeability. A solution containing 5% normal serum was used to block against nonspecific staining for 1 hour at room temperature. After the above steps, anti–SNAP–25 (rabbit, 1:5000; Sigma), anti–SV2 (goat, 1:200; Santa Cruz Biotechnology) and anti–TRPV1 (goat, 1:200; Santa Cruz Biotechnology) antibodies were added to the cell cultures and incubated overnight at 4°C. Secondary antibodies conjugated to Alexa Fluor–488 or –594 (Invitrogen, USA) were then used. Nucleus counter–staining was performed by mounting with anti–fade reagent containing DAPI (Invitrogen, USA). The coverslips containing neurons were viewed under an inverted microscope and a confocal microscope (Olympus, Japan) to detect IF. In addition, Nissl staining was used to calculate the percentage of SNAP–25–positive and TRPV1–positive neurons in cells cultured for different periods of time. Ten images from random positions on 5 coverslips representing each group were taken for quantitation.

To identify colocalization between TRPV1 and cleaved SNAP–25 or BoNT⁄A, three–week–old cultured DRG neurons were first pre–treated with 1 nmol⁄l of BoNT⁄A(pure BoNT⁄A, 150kDa, Metabiologics, Inc., Madison, USA) and then the toxin was removed after 60 min or 24 hours of treatment. The cultures were washed twice with 0.01 M PBS and fixed with 4% paraformaldehyde for 30 min. Double–labeling of TRPV1 with either cleaved SNAP–25 (mouse anti-cleaved SNAP–25, 1:200, Research & Diagnostic Antibodies, North Las Vegas, USA) or BoNT⁄A (rabbit anti-BoNT⁄A, 1:8000, Metabiologics, Inc., Madison, USA) was then assessed. An inverted immunofluorescence microscope was used to examine the colocalization of TRPV1 with cleaved SNAP-25 or BoNT⁄A.

### Western blot (WB) and co-immunoprecipitation

Three–week–old cultured DRG neurons were used for BoNT⁄A treatment and TRPV1 antibody interference experiments.

WB was used to determine the expression of SNAP–25, SV2A and TRPV1. Cultured DRG neurons were harvested using RIPA buffer (NaCl, 150 mM; Tris–base, 50 mM; EDTA, 2 mM; SDS, 0.1%; and Triton X–100, 1%; with fresh proteinase inhibitor). Cell extracts were then sonicated briefly and centrifuged. Supernatants were used for protein concentration assays and stored at –20°C for WB assays. Twenty micrograms of total protein was loaded onto 12.5% pre–cast gels (Bio-Rad, USA) for SDS–PAGE and WB analysis. Anti–SNAP-25 (1:6000), anti–SV2A (1:500) and anti–TRPV1 (1:500) antibodies were used to probe specific proteins. The positive protein bands were developed using chemiluminescence and imaged with a Bio–Rad Image System.

The specificities of both BoNT⁄A and TRPV1 were tested prior to this study. Two processes were used to confirm antibody specificities; 1) different concentrations of the antibodies were pre-incubated with blocking peptides and 2) primary antibodies were eliminated as negative controls. Finally, anti-BoNT⁄A at 1:8000 for immunofluorescent staining and at 1:15000 for WB, anti–TRPV1 at 1:200 for immunofluorescent staining and at 1:500 for WB was chosen.

### Co–immunoprecipitation and interactions between TRPV1 and BoNT⁄A

Three–week–old cultured DRG neurons were used for TRPV1 and BoNT⁄A co–immunoprecipitation and interaction assays. For co-immunoprecipitation studies, 1 nmol⁄l of BoNT⁄A (pure BoNT/A, 150 kDa, Metabiologics, Inc. USA) was added to cell cultures for efficient intoxication (Coffield and Yan, 2009). After 24 hours of exposure to BoNT⁄A, the membrane preparations (protein concentration: 500–700 μg⁄ml) were incubated with either anti–TRPV1 (goat anti-TRPV1 antibody, 5 μg⁄ml, Santa Cruz Biotechnology, Dallas, USA) or anti–BoNT⁄A (rabbit anti–BoNT⁄A antibody, 5 μg⁄ml, Metabiologics, Inc., Madison, USA) at 4°C on a rotator overnight. Membrane proteins were extracted using a Native Membrane Protein Extraction Kit (Calbiochem, USA). Protein–A agarose beads (20 μl, 25%) were added to the preparation and incubated for another 4–5 hours. The preparation complex was washed 3 times with Ralie Blot buffer (Bethyl Laboratory, Inc., USA). Then, the beads were resuspended in SDS–PAGE sample buffer (Bio-Rad, USA), followed by boiling for 5 min. The supernatant was loaded onto gels for SDS–PAGE. A concentration of normal bovine serum (BSA) equal to that of the antibody was loaded as a control. The protein was transferred onto a PVDF membrane (Amersham Hybond–P, GE Healthcare, USA), and then, either an anti–BoNT⁄A or an anti–TRPV1 antibody was used to probe the corresponding protein. The immunoreactive bands were visualized using chemiluminescent methods.

Another batch of DRG cultures were plated at 0.5x10^6^ /ml in 24–well plates and cultured for 3 weeks. Neurons were fixed with 4% paraformaldehyde for 30 min at room temperature. Anti–BoNT/A and anti–TRPV1 primary antibodies were added to the cells and incubated at 4°C overnight. Afterwards, secondary antibodies conjugated to either Alexa Fluor 488 or Alexa Fluor 594 were used for the immunofluorescent detection of TRPV1 and BoNT⁄A.

To examine the functional interaction between TRPV1 and BoNT⁄A, DRG cultures were pretreated with anti–TRPV1 antibody for 0, 1 and 2 hours before BoNT⁄A exposure. Then, the percentage of cleaved SNAP–25 (out of the total SNAP-25) after 24 hours of exposure to BoNT⁄A was determined by quantification of WB bands using Quantity One software (Bio-Rad Image System). The concentrations of TRPV1 antibody used to treat cells were 1:100 (200 ng⁄ml), 1:500 (100 ng⁄ml) and 1:1000 (20 ng⁄ml). Cleaved SNAP–25 was identified by the appearance of dual bands at approximately 25 kDa in 12.5% Criterion precast gels.

### Statistical analysis

All of the data for IF staining were derived from 10 random microscopic fields observed in 4 wells from the same group. The data for densitometry of WB bands were obtained from three separate experiments. The values are expressed as the mean ± SE. Statistical analysis for comparison of mean values was performed by one–way ANOVA followed by Tukey's multiple comparison test (GraphPad Prism 5.0, USA), and *P* < 0.05 was considered statistically significant.

## Results

### Expression of SNAP–25, SV2 and TRPV1 in cultured mouse embryonic DRG neurons

**Fig 1:**
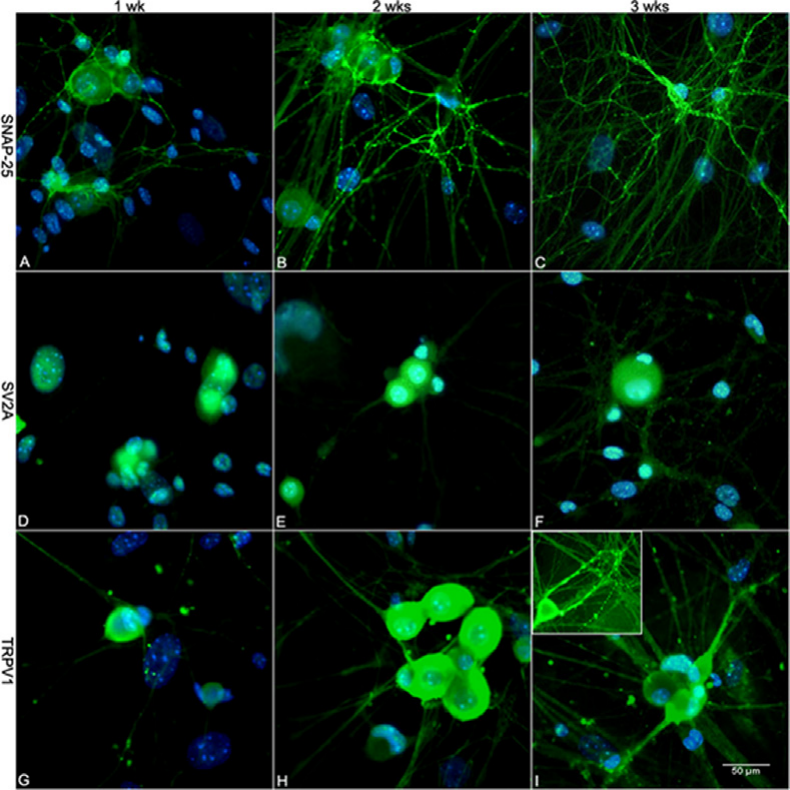
Immunofluorescence of SNAP-25, SV2A and TRPV1. 1A–1C: SNAP-25-positive neurons were observed in one-week-old DRG cultures, and positive staining was located both in the periphery of the cytoplasm and neuronal processes. The staining pattern did not change except that the number of SNAP-25-positive cells increased with longer culture periods. 1D–1F: Compared with the IF staining of SNAP-25, the positive staining for SV2A was mainly distributed in the cytoplasm and was less highly expressed along neuronal processes until 3 weeks of culture. Moreover, weak and scattered IF positivity for SV2A was observed in nuclei in the cells with shorter culture periods. 1G–1I: Much like SNAP-25, TRPV1 was also expressed in cultured DRG neurons after 1 week of culture. Initially, the positivity mainly appeared around the surface of the cell body. In the 2- and 3-week cultures, the positive immunofluorescence became much stronger. Obvious immunoreactivity for TRPV1 was observed in both the cytoplasm and processes when cells were cultured for up to 3 weeks, especially along the neuronal processes, as shown in the inset in 1I (bar = 50 μm).

**Fig 2:**
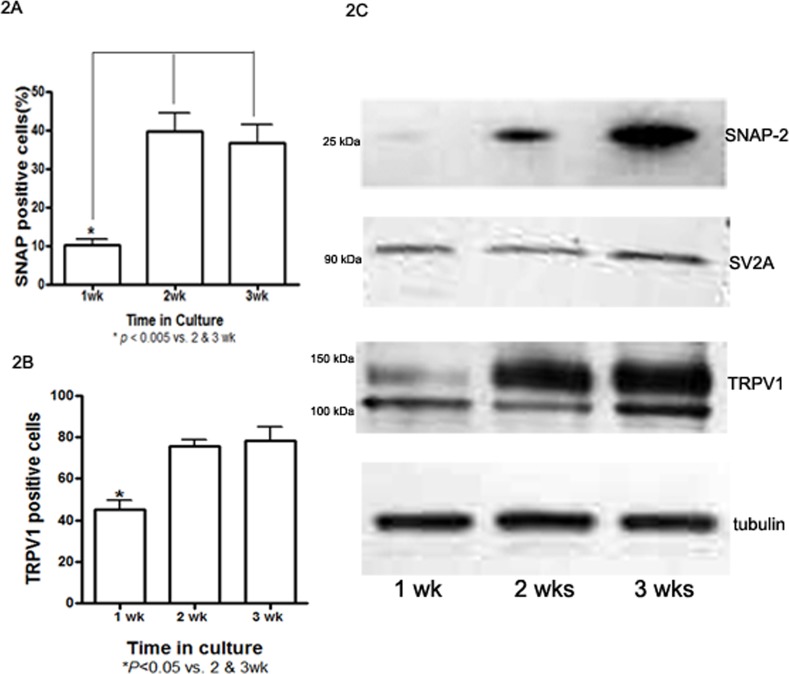
Expression of SNAP-25, SV2A and TRPV1 by SDS-PAGE and western blot. 2A: The distribution of SNAP-25 IF-positive neurons as a percentage of DAPI positive cells was dramatically increased after 2 weeks of culture. 2B: The percentage of TRPV1-positive neurons showed a similar pattern to that observed for SNAP-25. 2C: WB assays show that SNAP-25, SV2A and TRPV1 are strongly expressed in the extracts from 3-week-old DRG cultures, which confirms that 3-week-old cultured DRG neurons are a good cell model for studying BoNT/A action. 20 μg protein/lane; tubulin was used as a loading control.

Cultured embryonic DRG neurons express the structural machinery necessary for BoNT⁄A activity, including its membrane binding protein SV2 and target protein SNAP–25. First, the expression and distribution of SNAP–25 protein in cultured DRG neurons were determined by immunofluorescent staining (Fig 1A–1C). The percentage of SNAP–25 positive neurons was calculated based on DAPI counterstaining. The percentage of SNAP–25 positive neurons was only 10.18% ± 2.17% after the first week of culture. Interestingly, when the culture period reached two and three weeks, the percentage of SNAP–25–positive neurons clearly increased to 39.73% ± 9.60% and 36.73% ± 9.40%, respectively (*P* < 0.005 vs. one–week-old neurons, Fig 2A). In addition, the distribution of SNAP–25 in cultured DRG neurons varied between cells cultured for different periods of time. For example, in one–week–old DRG neurons, SNAP-25 immunoreactivity mainly appeared in the soma, with very faint staining in the processes. After two weeks in culture, definite staining in the neuronal processes was observed, and in three–week–old DRG cultures, very strong immunoreactivity was observed in both the neuronal processes and the periphery of the soma. Thus, three–week–old cultured DRG neurons expressed clear SNAP–25 immunoreactivity (Figs 1 & 2). The results from the WB analysis of SNAP–25 were consistent with the IF staining in that stronger expression also appeared in 3–week–old DRG cultures (Fig 2). Our previous data showed that three–week–old cultured DRG neurons were also susceptible to BoNT⁄A (data not presented here), which indicates that three–week–old cultured DRG neurons are a suitable model for BoNT⁄A–related research. Similar to SNAP–25, positive IF staining for SV2A was also observed after one week of cell culture, but it was mainly distributed in the neuronal cytoplasm and nuclei. IF positivity along neuronal processes was clearly observed only in the three–week–old cultured DRG neurons, although the cytoplasmic and nuclear positive staining was the same across the groups (Fig 1D–1F).

The distribution and expression of TRPV1 in cultured embryonic DRG neurons exhibited patterns very similar to those of SNAP–25, i.e., cultured DRG neurons also expressed TRPV1 as early as after one week of culture. At first, very scattered TRPV1–positive neurons were identified by their brightly stained somas. When the culture time reached two weeks, clear TRPV1 immunoreactivity appeared in the soma, and it was primarily distributed along the membrane and in neurites. The percentage of TRPV1 positive neurons increased in cells cultured for three weeks (Fig 1G–1I). Not surprisingly, most of the more strongly labeled TRPV1–positive neurons belonged to cells with small diameters. In addition, the band for TRPV1 was detected at approximately 110 kDa in the extracts of two to three–week–old DRG neuron cultures (Fig 2).

Based on these results showing the expression and distribution of SNAP–25, SV2A and TRPV1 in cultured DRG neurons, three–week–old embryonic DRG neuron cultures were used for further experiments.

### Interaction between TRPV1 and BoNT⁄A and colocalization with cleaved SNAP–25

**Fig 3:**
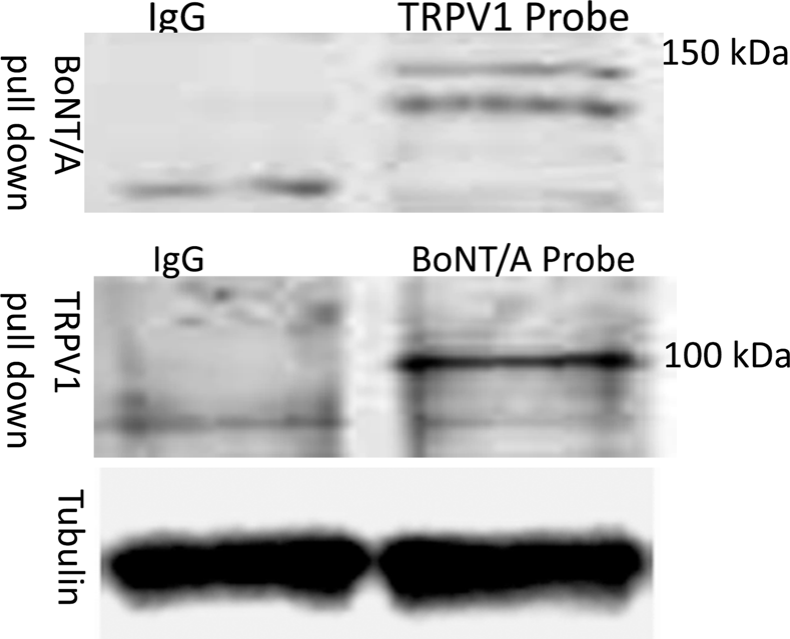
Co-immunoprecipitation and WB of TRPV1 and BoNT/A. After 1 hour of 1nmol of BoNT/A treatment in 3-week-old DRG neuron cultures, the membrane protein extracts were incubated with either anti-BoNT/A antibody or anti-TRPV1 antibody overnight at 4°C and the precipitated proteins of interested were probed by western-blot using the cognate antibody (Top and Middle panels). Tubulin was used as a loading control (Lower panel).

**Fig 4:**
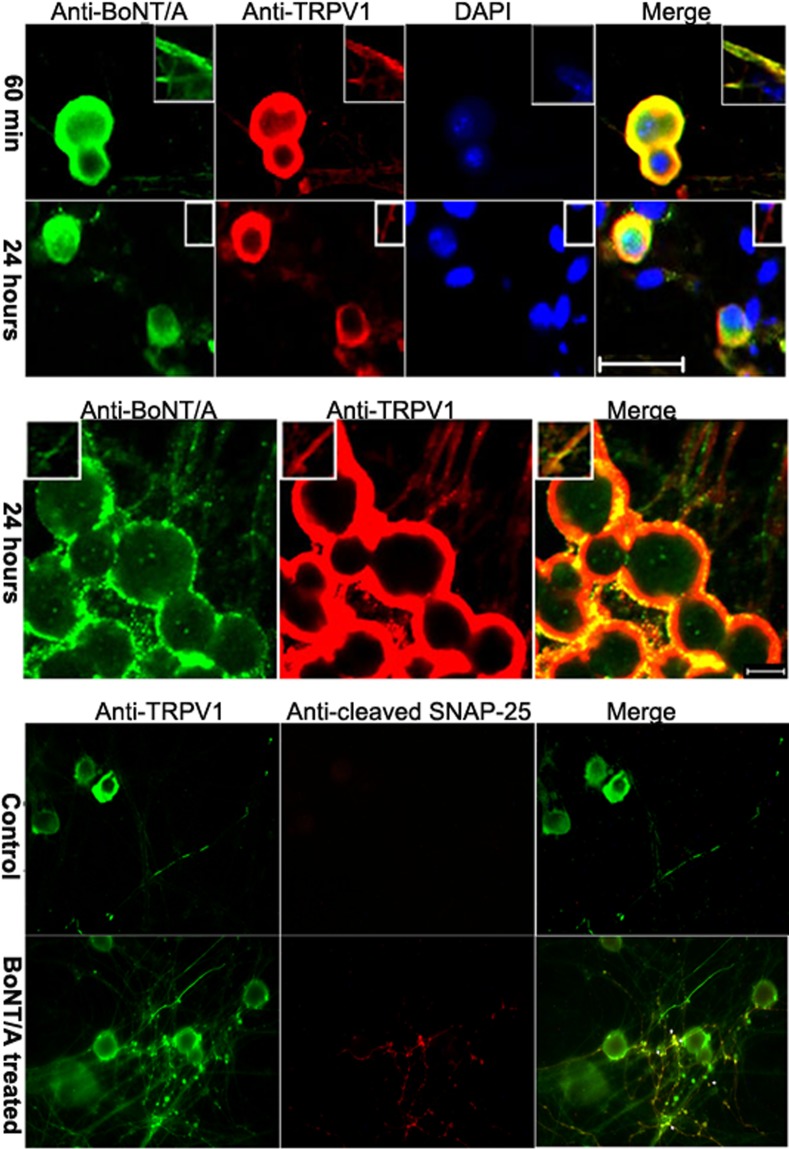
Immunofluorescent colocalization of TRPV1 with BoNT/A or cleaved SNAP-25. Top-panel: After cultured DRG neurons were exposed to BoNT/A for 60 min, BoNT/A immunoreactivity was observed to colocalize with TRPV1 in the membranes of both soma and neurites (inset). Middle-panel: BoNT/A IF reactivity moved into the cytoplasm when the exposure time was increased to 24 hours, and the double labeling of BoNT/A and TRPV1 appeared partially co-localized; meanwhile, the IF double labeling was not easily observed along neuronal processes in these cells (inset). Lower-panel: Under microscope, cleaved SNAP-25 was observed to be distributed in a punctate pattern along the cell membrane and was colocalized (dotty) with TRPV1 after DRG neurons were treated with BoNT/A for 24 hours (bar = 50 μm).

An interaction between BoNT⁄A and TRPV1 was revealed using co–immunoprecipitation and WB assays of DRG neuronal membrane extracts. First, the membrane extracts from three–week–old cultured DRG neurons were pre-treated with 1 nmol⁄l of BoNT⁄A and then incubated with either anti–BoNT⁄A or anti–TRPV1 antibodies. Next, the samples were probed with either anti–TRPV1 or anti–BoNT⁄A antibodies, respectively. Fig 3 displays co–immunoprecipitated pellets that contain both TRPV1 and BoNT⁄A, which were pulled down using either the anti–TRPV1 or the anti–BoNT⁄A antibody. Double–labeling of TRPV1 and BoNT⁄A (merged images) demonstrated that the two proteins are highly colocalized on the neuronal membranes of both soma and neuronal processes when stained immediately following 60 min of exposure to BoNT⁄A. However, after 24 hours of treatment with BoNT/A, the colocalization was observed only on soma membranes and not on neuronal processes, while the IF expression of BoNT⁄A decreased (Fig 4). These results suggest that BoNT⁄A interacts with surface TRPV1 on the membrane of DRG neurons via a relationship between TRPV1 and the binding protein for BoNT⁄A.

Furthermore, we provide evidence for the colocalization of TRPV1 with cleaved SNAP–25, the target protein of BoNT⁄A action. One of the interesting phenomena observed at this time was that TRPV1 was still positioned on the membrane while the positive staining for BoNT⁄A translocated into the cytoplasm (Fig 4). Visualization of TRPV1 with cleaved SNAP–25 revealed that cleaved SNAP–25 was present in a punctate or dotted pattern colocalized with TRPV1 along the neuronal processes (Fig 4).

**Fig 5:**
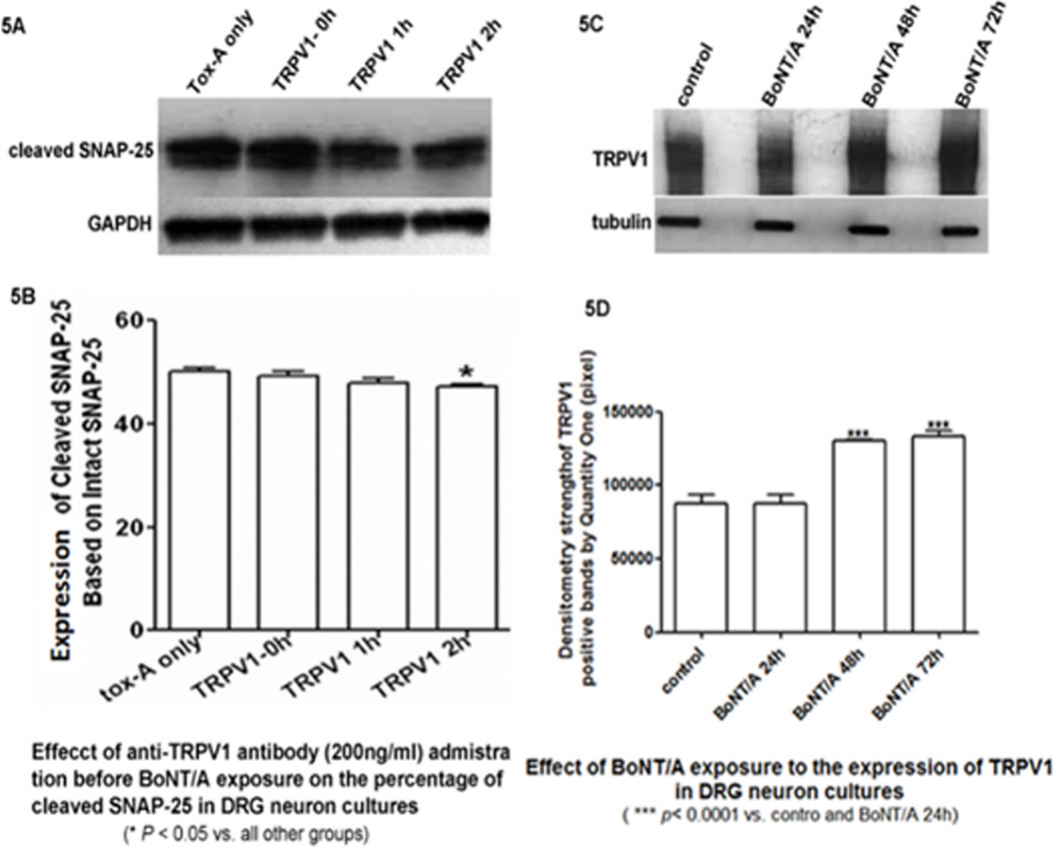
Functional interaction between BoNT/A and TRPV1 in cultured DRG neurons. 5A, 5B. The influence of anti-TRPV1 antibody applied before BoNT/A exposure on the percentage of SNAP-25 cleaved by BoNT/A. 5C, 5D. The effects of different exposure times of BoNT/A on the expression of TRPV1 in DRG neuron membrane extracts, as analyzed by WB.

Finally, experiments were performed to reveal a functional relationship between TRPV1 expression and the toxicity of BoNT⁄A. These experiments were based on the structural colocalization of TRPV1 with BoNT⁄A and cleaved SNAP–25 (because we did not use FRET, we cannot say that we observed an “interaction”). We examined the relationship between TRPV1 expression and the functional action of BoNT⁄A. Various titers of anti–TRPV1 antibody [1:100 (200 μg⁄ml), 1:500 (100 ng⁄ml), and 1:1000 (20 ng⁄ml)] were added to cultured DRG neurons prior to exposure to BoNT⁄A. The appearance of a slightly lower band indicating cleavage of SNAP–25 in WB analyses indicated the presence of functional BoNT⁄A. The change in the ratio of cleaved SNAP–25 to total SNAP–25 was calculated. It was found that the percentage of cleaved SNAP–25 decreased after anti–TRPV1 antibody was added at 1:1000 (200 ng⁄ml) 2 hours prior to BoNT⁄A intoxication. These data indicate a type of functional synergy between TRPV1 and BoNT⁄A (Fig 5A and 5B). Moreover, 48∼72 hours of incubation of cultured DRG neurons with 1 nmol/⁄ BoNT⁄A resulted in an increase in the expression of TRPV1 up to 30%∼40%, while there was no change after 24 hours of BoNT⁄A pretreatment. These data provide further support for the modulating effects of TRPV1 on BoNT⁄A activity and the dynamic alteration of TRPV1 expression by BoNT⁄A (Fig 5B and 5C).

## Discussion

Although it has been reported that BoNT⁄A alters the expression of TRPV1 [13–15] and that capsaicin can attenuate the paralysis induced by BoNT⁄A, the interaction between TRPV1 and BoNT⁄A has remained unclear. This study presents several lines of evidence to support the existence of such an interaction. The results showed that TRPV1 colocalizes with BoNT⁄A in addition to cleaved SNAP–25, the modified target protein of BoNT⁄A action. Interestingly, TRPV1 colocalized with BoNT⁄A on the cell membrane after the initial 60 min of BoNT⁄A exposure, but the co–localization was invisible after 24 hours of toxin treatment. This phenomenon might indicate that an interaction between TRPV1 and BoNT⁄A occurs only when BoNT⁄A binds to its receptors on the cellular membrane. However, only a scattered membrane pattern of BoNT⁄A was notable at 24 hours of incubation when most of the toxin had been endocytosed. The colocalization of TRPV1 and BoNT⁄A in cytoplasm was not observed by double–labeling IF. Interestingly, some punctate colocalization of TRPV1 and cleaved SNAP–25 was observed at the 24 hours exposure of toxin. Take altogether, these data suggest that the relationship of TRPV1 and BoNT⁄A is multi–faceted involving membranous and cytoplasmic interactions. Previous morphological studies suggested that alteration or inhibition of ganglioside would affect the sensitivity and immunoreactivity of TRPV1 by disturbing membrane lipid raft in sensory ganglion cells [19,20]. Trisialoganglioside 1b (GT1b), one of the major gangliosides in neuronal membrane, has been shown to be the low affinity binding receptor of BoNT⁄A. Thus, the colocalization and co–precipitation of TRPV1 and BoNT⁄A within 60 min of toxin treatment via a ganglioside lipid raft interaction might be hypothesized from this study. Like SNAP–25, TRPV1 may act as a synaptic protein as well [9]. Therefore, the colocalization of TRPV1 and cleaved SNAP–25, the resultant protein of BoNT⁄A action, might be regarded as an indication of an indirect interaction of TRPV1 and BoNT⁄A.

Interestingly, pretreatment of cultured DRG neurons with BoNT⁄A increased the expression of TRPV1 and applying anti–TRPV1 antibodies before toxin exposure decreased the percentage of cleaved SNAP–25. All of these findings indicate that TRPV1 interacts structurally with BoNT⁄A and that this interaction functionally alters both toxin activity and TRPV1 expression.

The basic toxic action of BoNT⁄A is characterized by the appearance of cleaved SNAP-25, one of the SNARE [SNAP (Soluble NSF Attachment Protein) Receptor] proteins. Since the initial use of BoNT⁄A as a therapeutic agent in the 1970s, the scope of its medical uses has grown substantially. The efficacy and safety of BoNT⁄A as a treatment for different pain syndromes, such as tension headaches, migraines, chronic lumbar pain, and myofascial pain, have been demonstrated both clinically and experimentally [2]. The inhibition of the release of pain-related peptides (e.g., CGRP and substance–P) from the afferent terminals of sensory neurons has been confirmed experimentally to be one of the major mechanisms by which BoNT⁄A confers pain relief [21,22]. However, the exact anti–nociceptive mechanisms through which BoNT⁄A provides pain relief are the subject of ongoing investigation.

There is no doubt that the structural impairment and dysfunction of SNAP–25 that is induced by BoNT⁄A is part of the mechanism that inhibits CGRP release and leads to pain alleviation. More recently, researchers have found that TRPV1 activation increased CGRP release and that BoNT⁄A was able to block this process [23]. These findings suggest the possibility of a structural or functional interaction between TRPV1 and BoNT⁄A. Evidence from other studies also supports the existence of this interaction: 1) the activation of TRPV1 protects the mouse neuromuscular junction against neuroparalysis by BoNT⁄A [24]; 2) BoNT⁄A modulates the expression of TRPV1 when locally injected into the urinary bladder subepithelium [13,14]; and 3) the decrease in suburothelial TRPV1 is correlated with a reduction in patients' pathological sensation of urgency [13,25]. These results suggest that further investigation is needed to clarify the physical interaction between TRPV1 and BoNT⁄A.

TRPV1 is one of the most widely studied nociceptors to date and has been used as a therapeutic target molecule. TRPV1 is a noxious sensor[26–28], and it might also be involved in many other pathological processes in addition to nociception. Structurally, TRPV1 subunits have six transmembrane (TM) domains, including intracellular N– (containing 6 ankyrin–like repeats) and C–termini and a pore region between TM5 and TM6 that contains sites that are important for channel activation and ion selectivity. The N– and C–termini have residues and regions that are sites for phosphorylation and dephosphorylation and which regulate TRPV1 sensitivity and membrane insertion. Based on its complicated chemical structure, it is reasonable to hypothesize that TRPV1 might interact with other membrane and intracellular molecules. An interaction between TRPV1 and calmodulin, a SNARE–associated protein similar to Snapin and Synaptotagmin–9, has been described in the literature [29,30]. Its coexpression with other membrane proteins, such as the voltage–gated potassium channel Kv1.4 [31], the cannabinoid receptor CB1 [31,32] and PAR2 [33], has also been described.

Uptake of BoNT⁄A at the nerve terminal occurs via synaptic vesicle endocytosis and has been shown by others to be mediated by the binding of BoNT⁄A to its specific membranous components: the high affinity binding protein (SV2) and low affinity receptor ganglioside (GT1b) [34, 35]. Both SV2 and GT1b are widely distributed both in the CNS and PNS [36–39]. Therefore, it is likely that BoNT⁄A gets into the sensory terminals by the same routes. Recently, evidence has emerged that lipid raft activity triggered by gangliosides played a role in the activation of TRPV1 and the depletion of ganglioside by inhibiting ganglioside synthase decreased the expression of TRPV1 and its activation by capsaicin [20,40]. Thus, it is reasonable to postulate that the binding of BoNT⁄A to GT1b might interrupt the lipid raft activity related to TRPV1, thereby inhibiting the activation of TRPV1. SV2 is a ubiquitous, integral membrane glycoprotein required for calcium–stimulated exocytosis [41]. On the other hand, TRPV1 has also been reported as a synaptic protein involved in vesicle recycling [9]. Although there is limited detailed information about the two vesicular proteins in PNS, based on the results of this current study, the possibility exists that BoNT⁄A may bind to TRPV1. Further study is warranted to explore this possibility.

The target protein for BoNT⁄A intoxication, SNAP–25, also belongs to the synaptic membrane protein family [42]. At the beginning of this study, we showed that the presence of these structural components (SV2 and SNAP–25) was necessary for BoNT⁄A intoxication in mouse embryonic DRG neuron cultures. Then, we showed by immunofluorescence that TRPV1 colocalizes not only with BoNT⁄A but also with cleaved SNAP–25. The colocalization of TRPV1 with BoNT⁄A suggests that BoNT⁄A interacts (either directly or indirectly) with TRPV1 when it reaches sensory terminals. The binding receptor for BoNT⁄A might somehow interact with TRPV1. The colocalization of TRPV1 with cleaved SNAP–25 further suggests that TRPV1 is a structural protein on the synaptic membrane, similar to SNAP–25, which has also been suggested by others [9]. The interaction between TRPV1 and BoNT⁄A was further confirmed by co–immunoprecipitation in the present study. Based on the above experiments, the anti–nociceptive effects of BoNT⁄A might be explained by the structural interaction between BoNT⁄A and TRPV1. More recently, several studies have reported that TRPV1 functionally interacts with BoNT⁄A, for example, capsaicin, a TRPV1 agonist, inhibits BoNT⁄A–induced paralysis[23], and BoNT⁄A decreases the expression of meningeal TRPV1 in the context of neuropathic pain [15]. However, the results from the analysis of TRPV1 expression following treatment with BoNT⁄A in DRG cultures showed a different pattern. TRPV1 expression was not changed 24 hours after BoNT⁄A pretreatment but was slightly increased at 48∼72 hours after pretreatment of the DRG cultures with 1 nmol⁄l of BoNT⁄A. A possible reason for this difference could be the dynamic changes in TRPV1 expression observed under the continuous application of BoNT⁄A. To identify whether the structural interaction between BoNT⁄A and TRPV1 affects the toxicity of BoNT⁄A, an anti–TRPV1 antibody was applied to sensory neurons before BoNT⁄A treatment. Because the light chain (LC) of BoNT⁄A is a Zn^2+^–dependent protease that cleaves approximately 9 amino acids off of the SNAP–25 molecule, two parallel bands appeared at approximately 25 and 24 kDa in the WB following BoNT⁄A intoxication. The upper band is intact SNAP–25, and the lower band is cleaved SNAP–25. The changes in the density ratio between the lower band and the sum of the lower and upper bands were regarded as evidence that anti–TRPV1 protected against the toxicity of BoNT⁄A. We found that the percentage of SNAP–25 that was cleaved by BoNT⁄A was decreased when 1:1000⁄ml (200 ng⁄ml) of anti–TRPV1 antibody was added to the cultured DRG neurons 2 hours before exposure to BoNT⁄A. This evidence strongly indicates that BoNT⁄A functionally interacts with TRPV1. The functional inhibition by the presence of the TRPV1 antibody diminishes the cleavage of SNAP–25 by BoNT⁄A, which prevents the release of vesicular contents.

The available data suggests that the anti-nociceptive effects of BoNT⁄A are achieved via the inhibition of pain–related neuropeptide release, which is likely a result of its cleavage of SNAP-25. However, the structural and functional interactions between BoNT⁄A and TRPV1 represent another important potential anti–nociceptive mechanism for BoNT⁄A. Based on all of the above data, more details regarding the interaction between TRPV1 and BoNT⁄A, especially in the context of their functional relationship, must be clarified in the future.

## Abbreviations

BoNTs: Botulinum neurotoxins
BoNT⁄A: Botulinum neurotoxin serotype A
TRPV1: Transient receptor potential vanilloid subtype 1
DRG: Dorsal root ganglion
IF: Immunofluorescent
WB: Western blot
